# Eucaine Anæsthesia

**Published:** 1900-04-07

**Authors:** 


### EUCAINE ANÆSTHESIA.

An interesting case is recorded by Mr. Vincent Jackson,[1] of Wolverhampton, in which he did suprapubic cystotomy under local anæsthesia, produced by the injection over the line of the incision of 40 minims of a seven-and-a-half per cent. solution of hydrochloride of eucaine (equal to about two-thirds of a grain of the drug). During the operation the forefinger of an assistant was passed into the rectum to raise up the floor of the bladder. When all was completed the towel was removed from the man's face, and he was asked if he felt anything, to which he replied, "I felt a finger in the fundament." Evidently he had not felt the operation—a good instance of the utility of eucaine anæsthesia.

[1] Lancet, March 31.

